# When urine sparkles: tumor lysis syndrome after immune checkpoint inhibitor therapy

**DOI:** 10.1590/2175-8239-JBN-2026-0013en

**Published:** 2026-08-24

**Authors:** Ana Rita Ramos, Rui Duarte, Rita Valério Alves

**Affiliations:** 1 Unidade Local de Saúde do Médio Tejo, Hospital de Torres Novas, Serviço de Nefrologia, Torres Novas, Portugal.; 2 Unidade Local de Saúde de Coimbra, Serviço de Nefrologia, Coimbra, Portugal.

Tumor lysis syndrome (TLS) is a life-threatening complication increasingly recognized with immune checkpoint inhibitors, although uncommon and potentially delayed^1,2^.

We report acute kidney injury in a patient with metastatic melanoma after ipilimumab (3 mg/kg) plus nivolumab (1 mg/kg). Twenty-two days later, urine sediment revealed pleomorphic, strongly birefringent crystals consistent with uric acid crystalluria (Figure 1). Laboratory findings fulfilled Cairo–Bishop criteria, with hyperuricemia (18.3 mg/dL), hyperphosphatemia (10.5 mg/dL), a >25% increase in serum potassium, and creatinine of 8.4 mg/dL. Differential diagnoses included drug-induced crystalluria and other causes of acute urate nephropathy.

**Figure 1:**
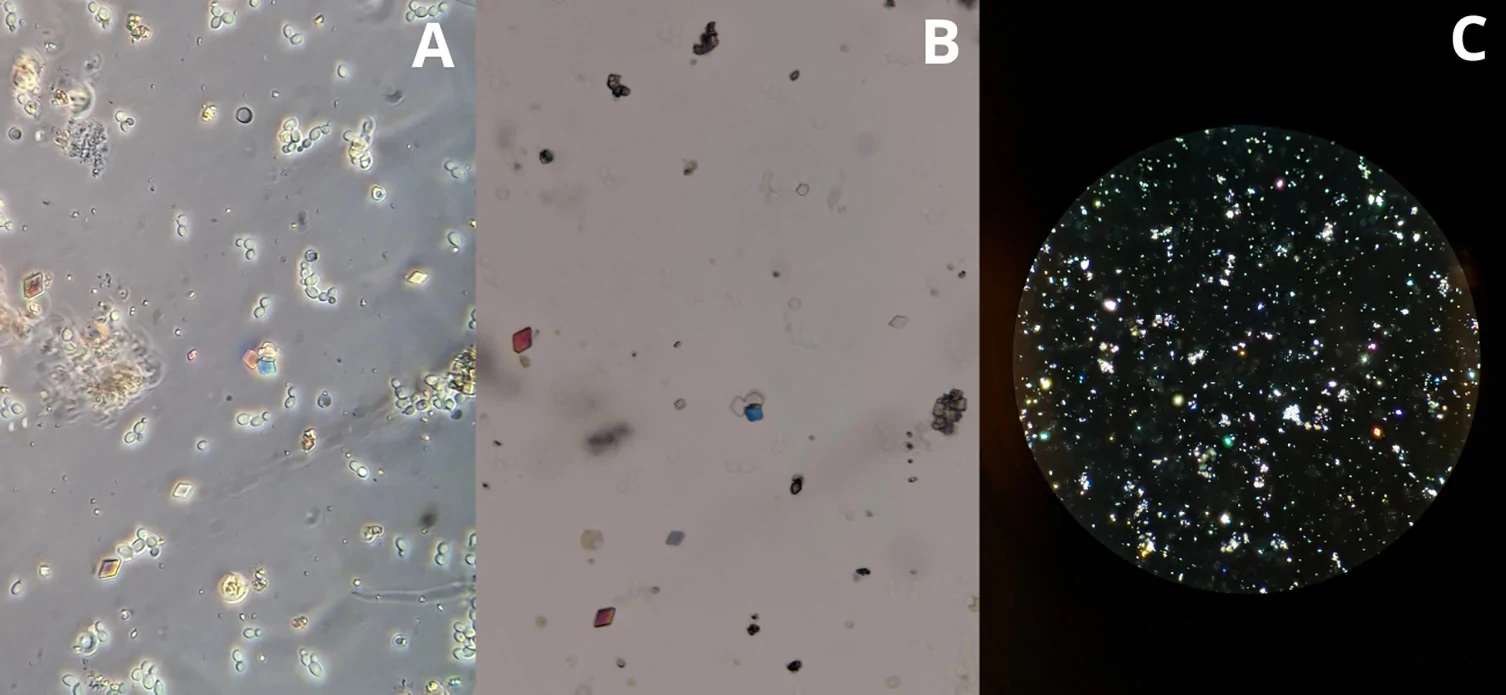
Urine sediment examination. (A, B) Bright-field microscopy showing abundant pleomorphic uric acid crystals. (C) Polarized light microscopy demonstrating intense birefringence, corroborating the diagnosis of tumor lysis syndrome (×400 magnification).

This image highlights urine sediment analysis as a rapid tool for early TLS recognition.

## Data Availability

Data are available from the corresponding author upon reasonable request.

## References

[1] Cairo MS, Bishop M (2004). Tumour lysis syndrome: new therapeutic strategies and
classification.. Br J Haematol..

[2] Wang L, Li X, Zhao B, Mei D, Jiang J, Duan J (2021). Immune checkpoint inhibitor–associated tumor lysis syndrome: a
real-world pharmacovigilance study.. Front Pharmacol..

