# Supplementary material for: When urine sparkles: tumor lysis syndrome after immune checkpoint inhibitor therapy
Source: J Bras Nefrol. 2026 Aug 24;48(4):e20260013. doi: 10.1590/2175-8239-JBN-2026-0013en (PMC13502950; doi:10.1590/2175-8239-JBN-2026-0013en)
Supplement: Translation

# Translation: Quando a urina brilha: síndrome de lise tumoral após terapia com inibidores de checkpoints imunológicos

**DOI:** 10.1590/2175-8239-JBN-2026-0013pt

**Authors:** Ana Rita Ramos, Rui Duarte, Rita Valério Alves

**Affiliations:** 1 Unidade Local de Saúde do Médio Tejo, Hospital de Torres Novas, Serviço de Nefrologia, Torres Novas, Portugal.; 2 Unidade Local de Saúde de Coimbra, Serviço de Nefrologia, Coimbra, Portugal.

A síndrome de lise tumoral (SLT) constitui uma complicação potencialmente fatal, cada vez mais reconhecida em associação com os inibidores de *checkpoint* imunológico, embora permaneça rara e possa apresentar-se de forma tardia^1,2^.

Descreve-se um caso de lesão renal aguda em um paciente com melanoma metastático após terapêutica com ipilimumab (3 mg/kg) e nivolumab (1 mg/kg). Vinte e dois dias após o início do tratamento, o exame do sedimento urinário revelou a presença de cristais pleomórficos, intensamente birrefringentes, compatíveis com cristalúria de ácido úrico (Figura 1). Os achados laboratoriais cumpriam os critérios de Cairo–Bishop, destacando-se hiperuricemia (18,3 mg/dL), hiperfosfatemia (10,5 mg/dL), aumento superior a 25% do potássio sérico e creatinina de 8,4 mg/dL. O diagnóstico diferencial incluiu cristalúria induzida por fármacos, bem como outras causas de nefropatia aguda por uratos.

**Figura 1:** Exame do sedimento urinário. (A, B) Microscopia de campo claro demonstrando abundantes cristais pleomórficos de ácido úrico. (C) Microscopia com luz polarizada evidenciando intensa birrefringência, sustentando o diagnóstico de síndrome de lise tumoral (aumento ×400).

Esta imagem evidencia o papel do exame do sedimento urinário como ferramenta rápida e acessível na identificação precoce da SLT.

## Data Availability

Os dados que sustentam os resultados deste estudo estão disponíveis mediante pedido razoável ao autor correspondente.
